# Neutrophil to Lymphocyte Ratio (NLR)—A Useful Tool for the Prognosis of Sepsis in the ICU

**DOI:** 10.3390/biomedicines10010075

**Published:** 2021-12-30

**Authors:** Alice Nicoleta Drăgoescu, Vlad Pădureanu, Andreea Doriana Stănculescu, Luminița Cristina Chiuțu, Paul Tomescu, Cristiana Geormăneanu, Rodica Pădureanu, Vlad Florin Iovănescu, Bogdan Silviu Ungureanu, Andrei Pănuș, Octavian Petru Drăgoescu

**Affiliations:** 1 Department of Anesthesiology and Intensive Care, University of Medicine and Pharmacy of Craiova, 200349 Craiova, Romania; alice.dragoescu@yahoo.com (A.N.D.); luminita.chiutu@gmail.com (L.C.C.); 2 Department of Internal Medicine, University of Medicine and Pharmacy of Craiova, 200349 Craiova, Romania; 3 Department of Urology, University of Medicine and Pharmacy of Craiova, 200349 Craiova, Romania; paul.tomescu@yahoo.com (P.T.); andrei.panus@umfcv.ro (A.P.); pdragoescu@yahoo.com (O.P.D.); 4 Department of Emergency Medicine and First Aid, University of Medicine and Pharmacy of Craiova, 200349 Craiova, Romania; cristiana.geormaneanu@umfcv.ro; 5 Department of Internal Medicine, Emergency Clinical County Hospital of Craiova, 200642 Craiova, Romania; zegheanurodica@yahoo.com; 6 Department of Gastroenterology, University of Medicine and Pharmacy of Craiova, 200349 Craiova, Romania; vlad.iovanescu@umfcv.ro (V.F.I.); bogdan.ungureanu@umfcv.ro (B.S.U.)

**Keywords:** neutrophil to lymphocyte ratio (NLR), sepsis, septic shock, presepsin

## Abstract

Sepsis is a life-threatening medical emergency induced by the body′s extreme response to an infection. Despite well-defined and constantly updated criteria for diagnosing sepsis, it is still underdiagnosed worldwide. Among various markers studied over time, the neutrophil to lymphocyte ratio (NLR) recently emerged as a good marker to predict sepsis severity. Our study was a single-center prospective observational study performed in our ICU and included 114 patients admitted for sepsis or septic shock. Neutrophil to lymphocyte ratio (NLR) is easy to perform, CBC being one of the standard blood tests routinely performed upon admission for all ICU patients. We found that NLR was increased in all patients with sepsis and significantly raised in those with septic shock. NLR correlates significantly with sepsis severity evaluated by the SOFA score (R = 0.65) and also with extensively studied sepsis prognosis marker presepsin (R = 0.56). Additionally, NLR showed good sensitivity (47%) and specificity (78%) with AUC = 0.631 (*p* < 0.05). NLR is less expensive and easier to perform compared with other specific markers and may potentially become a good alternate option for evaluation of sepsis severity. Larger studies are needed in the future to demonstrate the prognosis value of NLR.

## 1. Introduction

Sepsis is a life-threatening medical emergency induced by the body′s extreme response to an infection. Without an early and aggressive management, sepsis can rapidly lead to organ failure, tissue hypoperfusion, and death. Bacterial infections cause most cases of sepsis but it can also be a result of other infections such as viral, fungal, or parasitic infections. Sepsis-3 definitions for sepsis management were established in 2016 according to the Surviving Sepsis Campaign guidelines [1]. They define sepsis as “a life-threatening organ dysfunction caused by a dysregulated host response to infection” and septic shock as “a subset of sepsis in which underling circulatory and cellular/metabolic abnormalities are profound enough to substantially increase mortality” [1].

According to Sepsis-3 criteria’s, sepsis and septic shock are currently diagnosed as follows: sepsis—suspected or documented infection plus SOFA (Sequential Organ Failure Assessment) score ≥2; septic shock—sepsis plus vasopressor therapy needed to maintain MAP ≥65 mmHg plus lactate ≥2 mmol/L (18 mg/L) despite adequate fluid resuscitation [2]. However, despite these diagnosis criteria, the early evaluation of sepsis severity and prognosis is still imprecise and significant research is being performed to address this issue.

The neutrophil/lymphocyte ratio (NLR) was studied in order to provide an easier way to diagnose sepsis. This ratio can be calculated both from the absolute number of neutrophils and lymphocytes, and from their relative number. The importance of this report derives from the fact that physiological stress causes an increase in the number of neutrophils and a decrease in the number of lymphocytes. Sepsis stimulates lymphocyte apoptosis, so this ratio is increased in these cases. Septic shock causes a dramatic decrease in lymphocyte counts, so the NLR ratio increases significantly [3].

Presepsin is a 13 KDa polypeptide made by proteolytic cleavage of soluble forms of cluster of differentiation CD14 (sCD14). CD14 is present predominantly on most immune system cells, as well as cartilage, brain, liver, and intestinal cells [4]. To date, no clear correlation has been established between presepsin and NLR, but only between procalcitonin, WBC, or C-reactive protein [5]. It has not been established whether this easy-to-achieve parameter is superior to other markers for diagnosing sepsis and septic shock.

In one of our previous recent studies, we analyzed the value of presepsin as a marker for prognosis of sepsis severity and mortality risk and found significant results [6]. We therefore decided to continue our analysis of potential sepsis markers by evaluating the potential correlation between NLR and the severity of sepsis as well as presepsin and the SOFA score.

Our hypothesis is that the use of the simple neutrophil/lymphocyte ratio (NLR) may have a prognosis value similar to presepsin or the SOFA score in evaluating sepsis severity. The specific objective of this study was to determine whether NLR may be used as a prognosis factor in sepsis comparable to presepsin or the SOFA score.

## 2. Patients and Methods

This was a prospective single-center observational study performed in the ICU department of Craiova Emergency Hospital between October 2020 and June 2021.

Inclusion criteria for the study were: age ≥ 18, sepsis diagnosed by the SOFA score of pulmonary, abdominal, urinary, and surgical or unknown origin, as well as lactate levels ≥2 mmoL/L and need of vasopressors for mean arterial pressure (MAP) ≥65 mmHg, despite adequate volume resuscitations for patients with septic shock. Exclusion criteria were patients younger than 18 years old, pregnant females, immunocompromised patients (liver failure, auto-immune diseases, hematological cancer, organ transplant, AIDS), or those with terminal or severe illnesses (advanced or metastatic cancer, advanced congestive heart failure, stroke, coma). Before study enrollment, all patients (or close relatives if patient was unconscious or unable) were informed about the study and provided signed informed consent.

Of an initial sample of 173 patients with sepsis or septic shock admitted in our ICU department and evaluated for study enrollment, 59 were excluded due to various criteria (terminal or severe illness 28, patient refusal 18, immunocompromised 11, pregnant females 2). The remaining 114 patients were included in the study and, based on disease severity, were divided into two study groups: sepsis group—76 patients, and septic shock group—38 patients.

Personal medical history as well as clinical signs and symptoms were collected from all patients by qualified medical personnel. Complete clinical examination was completed at ICU admission. Glasgow Coma Scale/Score (GCS) was evaluated on the basis of the eye (4), verbal (5), and motor (6) standard criteria. Regular venous blood sampling was drawn upon admission for standard blood tests: hematology, biochemistry, arterial blood gases, serum lactate, as well as blood inflammation markers: C-reactive protein (CRP) and presepsin. As soon as possible after ICU admission, two pairs of blood cultures (aerobic and anaerobic) and various cultures from presumed infection sites were sampled before starting empiric antibiotic management. X-ray, ultrasound, or computed tomography tests were performed as needed [2]. Study parameter evaluation included NLR evaluation and measurement of presepsin values.

NLR increases rapidly in case of physiological stress, the growth time being approximately 6 h, thus being superior to the increase in the number of leukocytes or their deviation to the left. Normal NLR values are 1–3 [7,8]. It seems these values increase in proportion to the severity of sepsis, especially in septic shock.

Presepsin was analyzed using the method by PATHFAST^TM^ Immunoassay Analytical System, Mitsubishi Chemical Corporation, Tokyo, Japan (chemi-luminescent enzyme immunoassay—CLEIA, for the quantitative measurement of the presepsin levels in whole blood or plasma), using PATHFAST Presepsin test kits PF1201-K (LSI Medience Corporation, provided by Mitsubishi Chemical Europe GmBH, Düsseldorf, DE) [6,9]. Presepsin values are interpreted as follows [9]: <200 pg/mL—sepsis excluded, <300 pg/mL—systemic infection improbable, <500 pg/mL—sepsis probable, <1000 pg/mL—significant risk of severe sepsis, ≥1000 pg/mL—high risk of severe sepsis/septic shock equivalent to SOFA ≥8.

All study procedures involving human participants were performed in accordance with the ethical standards of the institutional and/or national research committee and with the 1964 Helsinki Declaration and its later amendments or comparable ethical standards.

Statistical tests and analysis were performed using the SciStat^®^ online statistical platform. Normal data distributions were evaluated by the D’Agostino-Pearson test. The parameters with normal distribution were compared by the Student *t*-test, while Mann–Whitney *U*-test was used as a non-parametric test. Parameter values were represented as mean ± SD (standard deviation) or median and 25%/75% quartile range. Correlations between study parameters were performed by Pearson *r* correlation analysis and receiver operating characteristics (ROC) curve analysis was performed with Area Under Curve (AUC) calculation and parameter cut-off values, as well as AUC comparison [6].

## 3. Results

The study included 114 patients admitted in the ICU from our hospital with sepsis diagnosis criteria according to Sepsis-3’s criteria [1]. Sepsis origin was pulmonary (42), urinary (27), surgical (18), abdominal (12), cutaneous (10), or unknown (5). Most of these patients had sepsis with or without organ failures (76) and the rest (38) were diagnosed with sepsis shock.

There were no significant differences between the two patient samples regarding age or sex. Hematology, biochemistry, and coagulation blood tests showed no significant differences between the two samples, except lower platelets for patients with septic shock. Although total lymphocyte count was similar in both groups, their ratio (%) was significantly lower in patients with septic shock (8.8% vs. 9.8%, *p* < 0.05). As expected, SOFA scores were elevated for all patients and significantly greater for those with septic shock. Additionally, patients with septic shock had a significantly lower GCS (11.2 ± 1.9 vs. 12.7 ± 1.7) and PaO_2_ (85 vs. 77 mm Hg), with higher need for mechanical ventilation, higher FiO_2_, and lower hypoxemic Horowitz index (PaO_2_/FiO_2_ ratio, 173 vs. 216, *p* < 0.05). Systemic inflammation marker ESR (erythrocyte sedimentation rate) was similar in both groups, while CRP (C-reactive protein) was elevated for patients with septic shock (118 vs. 133 mg/mL, *p* < 0.05). Presepsin was raised in all patients, but significantly higher in septic shock patients (1476 vs. 2403 ng/mL, *p* < 0.001). Sepsis-related death rate was 34% overall, 24% for sepsis patients and 55% for those with septic shock (*p* < 0.05). The most significant patient characteristics are presented below (Table 1).

**Figure 1:**
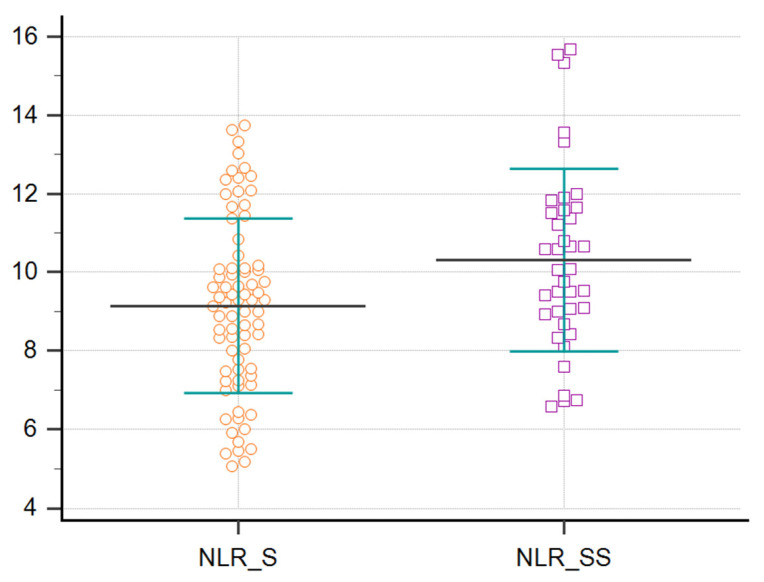
NLR values for patients with sepsis (NLR_S) and septic shock (NLR_SS). Data represented as mean, standard deviation (SD), and individual patient value markers.

Neutrophil to Lymphocyte Ratio (NLR) was subsequently calculated by dividing absolute neutrophil count to absolute lymphocyte count. All patients had higher than normal NLR values with an overall average of 9.53 ± 2.31. The values were significantly higher (*p* = 0.0126) for patients with septic shock (10.31 ± 2.32) compared with the sepsis group (9.15 ± 2.21), suggesting the potential value of NLR in assessing sepsis severity (Figure 1).

**Figure 2:**
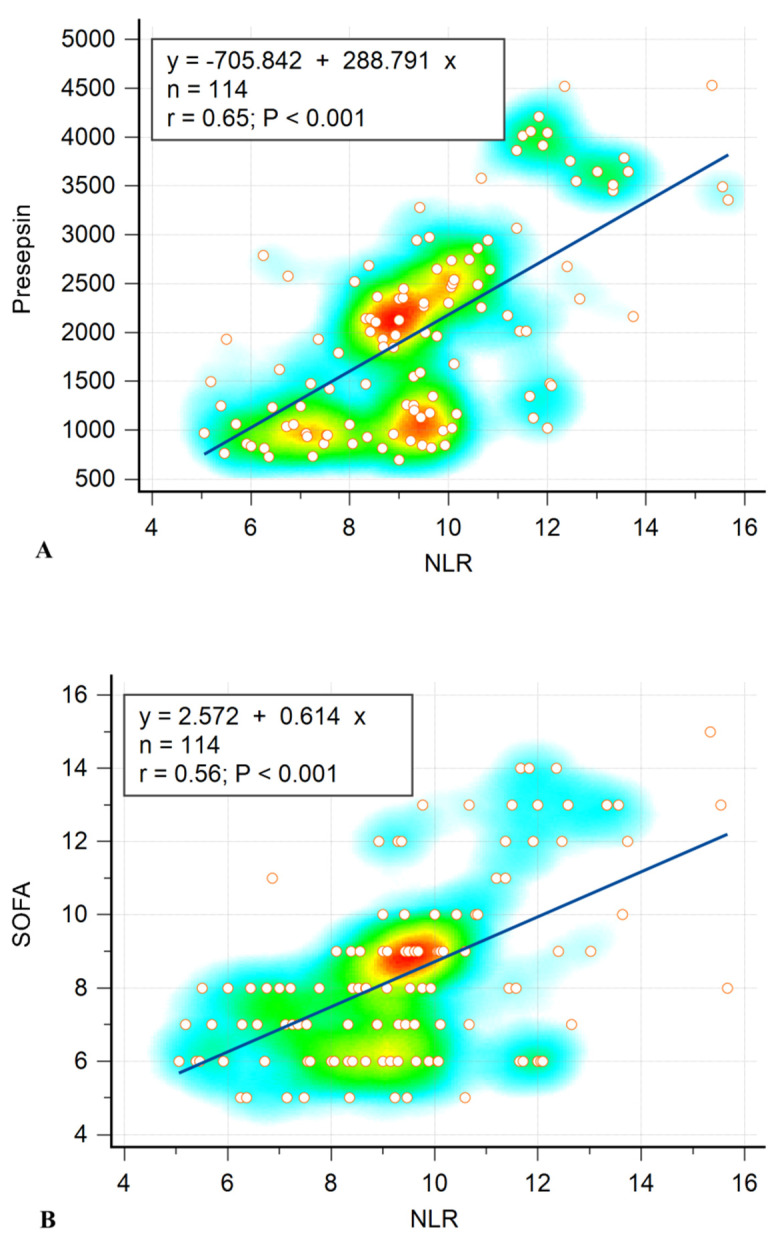
Correlations between NLR and Presepsin (**A**), NLR and SOFA (**B**). Data are presented as a scatter diagram with individual value markers, linear trendline, and heat-map. Correlation equation (y/x), correlation coefficient (r), and statistical significance (p) are provided within the graph.

Correlations between NLR and the severity and prognosis of sepsis were consequently evaluated. SOFA score and presepsin were chosen as adequate markers for this assessment as they are significantly modified in patients with severe forms of sepsis and widely used for prognostic purposes [9]. In our previous paper, we analyzed the correlations between the two markers and found a strong correlation between them (*r* = 0.88, 95% CI = 0.83–0.92, *p* < 0.001) [4]. As shown below (Figure 2), NLR correlated significantly with presepsin (*r* = 0.65, 95% CI = 0.53–0.74, *p* < 0.001) as well as the SOFA score (*r* = 0.56, 95% CI = 0.42–0.67, *p* < 0.001).

Receiver operating curve (ROC) analysis was performed to evaluate the prognosis value of NLR compared with the SOFA score and presepsin in sepsis severity assessment. As demonstrated previously [6], presepsin displayed very good sensitivity and good specificity (Sn = 79%, Sp = 63%) for identifying severe patients with a significant AUC = 0.726 (95% CI 0.635–0.806, *p* < 0.0001) for a cut-off value of 1932 ng/mL. Likewise, the SOFA score had good sensitivity, a lower specificity (Sn = 76%, Sp = 54%), and an AUC value of 0.651 (95% CI 0.556–0.738, *p* = 0.0057, criterion SOFA = 7). NLR presented a significant AUC, similar to that of the SOFA score (AUC = 0.631, 95% CI 0.536–0.720, *p* = 0.0159), for a cut-off value of 10.42, but a lower sensitivity (47%) with very good specificity (78%). This finding suggests that, unlike the SOFA score and presepsin, NLR may be more useful to easily identify patients with a more severe form of sepsis than those without, if calculated value is above 10.

**Figure 3:**
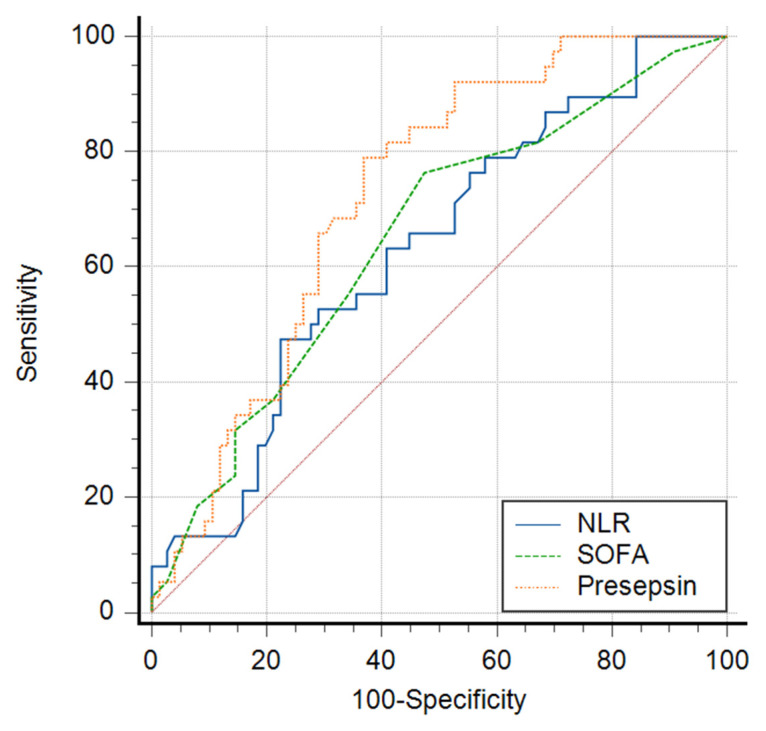
ROC curves comparison for NLR, SOFA score, and presepsin in septic shock. Data are presented as ROC curve for each parameter and 0.5 AUC line.

We finally performed a pairwise comparison of the ROC curves (Figure 3) that confirmed that presepsin is the better marker when compared with both the SOFA score (AUC difference = 0.075, *p* < 0.01) and NLR (AUC difference = 0.095, *p* < 0.01). However, NLR and SOFA score ROC curves comparison showed very similar results (AUC difference = 0.020, *p* = 0.75), which implies they may have similar prognostic values in detecting severe sepsis patients.

## 4. Discussion

Blood leukocytes have constantly been used in the past for diagnosing sepsis or septic shock. Patients diagnosed with sepsis or septic shock may have either leukocytosis or leukopenia, but many of them have a normal value of leukocytes [10]. Due to the fact that blood leukocytes are not therefore accurate in the diagnosis of sepsis or septic shock, several studies have tried to demonstrate that other parameters resulting from the leukocyte formula may be more useful. Neutrophil to lymphocyte ratio (NLR), which is the ratio of neutrophils/lymphocytes, recently emerged as such a parameter [3,6]. The fundamental mechanism of NLR increase is still uncertain, but it probably is induced by sepsis induced apoptosis of lymphocytes or due to some combination of endogenous cortisol and catecholamines, as they are known to elevate number of neutrophils while decreasing number of lymphocytes [11,12].

Neither the neutrophils or lymphocytes count offers reliable information about sepsis or septic shock. Our study showed that neutrophils were overall elevated, but similar in both patient groups. Similarly, total lymphocyte count decreased in all patients, but similar in both groups while their ratio (%) was significantly lower in patients with septic shock (8.8% vs. 9.8%, *p* < 0.05). These data confirm the previous findings in other similar studies [3].

Normal value of NLR is around 1–3 [4,7]. The increased values of NLR are correlated with the level of physiologic stress, especially in patients diagnosticated with septic shock. Thus, the NLR and its correlation with the severity of sepsis and septic shock was reviewed [3]. In our study, all patients had higher than normal NLR values with an overall average of 9.53 ± 2.31. The values were significantly higher (*p* = 0.0126) for patients with sepsis shock (10.31 ± 2.32) compared with the sepsis group (9.15 ± 2.21), suggesting the potential value of NLR in assessing sepsis severity, especially when above 10.

There are limited studies regarding the use of NLR for the diagnosis of sepsis and septic shock, though some of them have evaluated the importance of NLR in the diagnosis of diverse infections (e.g., appendicitis). These studies established that NLR is generally superior to the white blood cells count (WBC) [13,14]. Other studies revealed that NLR has comparable or superior performance compared with C-reactive protein (CRP), but is frequently inferior to procalcitonin regarding sepsis or septic shock [15].

We evaluated the correlation between NLR and sepsis severity markers and found a significant correlation with presepsin (*r* = 0.65, *p* < 0.001) as well as the SOFA score (*r* = 0.56, *p* < 0.001), which confirmed the potential importance of NLR as a prognostic tool in sepsis patients that may be similar to presepsin or the SOFA score.

Presepsin was shown to be very beneficial for the initial diagnosis of sepsis, as well as prediction of severity and mortality. Our prior study suggested the potential prognosis assessment of presepsin in patients with sepsis by being able to distinguish between patients with sepsis and septic shock, as well as in predicting sepsis-related mortality risk from ICU admission [5].

The results from previously studies demonstrate that the most practical parameter from the blood count cells might be the NLR, as it provides similar information about infection to the other serum markers like presepsin or severity scores such as SOFA, but is significantly easier to perform.

NLR was also used to estimate the outcomes of sepsis and septic shock management by Terradas et al. They showed that if the therapeutic management is effective, NLR will usually begin to decrease within a few days. If the NLR value remains high despite initiation of therapy, the prognosis is poor and the mortality risk is high [16,17]. It is likely that, if performed, daily monitoring of NLR would provide further useful prognostic data.

Several recent studies assessed the prognostic value of NLR in septic patients by ROC/AUC analysis. Liu et al. found an AUC of 0.695, but a quite high NLR value (≥23.8) reported as cut-off [18]. A similar study by Akilli et al. reported an AUC of 0.61 with NLR ≥11.9 as cut-off value [19] while Zhang et al. obtained an AUC of 0.718 [20]. Mandal et al. analyzed NLR value in predicting mortality and reported a very high AUC of 0.800 with NLR > 10 as optimal cut-off [21]. A more recent study from 2020 by El Said et al. evaluated both NLR and presepsin and found significant prognostic values: AUC = 0.890 with 92% sensitivity and 83% specificity at a 2100 ng/mL cut-off for presepsin and AUC = 0.758 with 75% sensitivity and 79% specificity for NLR = 17 [22].

In our study, NLR similarly displayed a significant AUC of 0.631, with a cut-off value of 10.42, but revealed a lower sensitivity (47%) with very good specificity (78%) while presepsin had a very good sensitivity (79%) and lower specificity (63%), AUC = 0.726 for a cut-off value of 1932 ng/mL, similar to the results of Ahmed et al. Our findings suggested that, unlike the SOFA score and presepsin, NLR may be more useful to easily identify patients with a more severe form of sepsis than those without if the calculated value is above 10. Moreover, NLR and SOFA ROC curves were very similar (AUC difference of only 0.020), which implies they may have similar prognostic values in detecting severe sepsis patients.

NLR is an easy parameter to perform, the complete blood count being one of the standard, mandatory tests for any hospitalized patient, so it can be widely used to assess the severity of sepsis or septic shock. Furthermore, using the cut-off value of 10, as obtained in our study and suggested by other authors [3], may help for a faster, mental calculation of the potential sepsis severity. Nevertheless, larger studies which will include more patients are needed to positively demonstrate the effectiveness of NLR in predicting the severity of sepsis, as well as assessing the risk of mortality in these patients.

Our study suggested that NLR might be a good prognostic tool. However, due to low sample size, which is a limitation of our study, a larger number of patients need to be evaluated to fully verify our findings.

## 5. Conclusions

In the fast-paced setting of sepsis management in the ICU, where patient prognosis assessment and adequate intensive treatment are vital, NLR, which is significantly less expensive, faster, and easier to perform compared with other sepsis-specific markers or severity scores, may potentially become a good alternate option for quick mental evaluation of sepsis patients due to its rapid and significant change that correlates with sepsis severity. However, since NLR lacks high sensitivity, further research is certainly required to confidently prove its effectiveness in predicting sepsis severity.

## Figures and Tables

**Table 1:** Study patients′ characteristics (most significant parameters selected). Data presented as mean ± SD or median and quartile range (25%–75%). (ns = not-significant, n/a = not applicable, s = sepsis, ss = septic shock, * = Fisher′s exact test, ^#^ = Mann-Whitney U test, ^+^ = Student t-test, WBC= white blood cells, NLR = Neutrophil to Lymphocyte Ratio, SOFA = Sequential Organ Failure Assessment, ESR = Erythrocyte Sedimentation Rate, CRP = C Reactive Protein, GCS = Glasgow Coma Scale).

Parameter	Total (*n* = 114)	Sepsis (*n* = 76)	Septic Shock (*n* = 38)	*p* (s/ss)
Age	71.25 ± 8.44	70.59 ± 8.64	72.55 ± 7.97	ns ^+^
Sex (M/F)	67/47	46/30	21/17	ns *
WBC (×10^3^/mm^3^)	16.3 (13.4–18.4)	16.4 (13.7–18.2)	16.2 (12.1–18.6)	ns ^#^
Neutrophils (×10^3^/mm^3^)	13.9 (11.3–16.1)	13.9 (11.4–16.0)	14.0 (10.4–16.5)	ns ^#^
Lymphocytes (×10^3^/mm^3^)	1.40 (1.20–1.79)	1.40 (1.20–1.80)	1.35 (1.13–1.68)	ns ^#^
Platelets (×10^3^/mm^3^)	140 (101–168)	143 (113–179)	125 (68–166)	0.031 ^#^
NLR	9.53 ± 2.31	9.15 ± 2.21	10.31 ± 2.32	0.0126 ^+^
Presepsin (ng/mL)	1968 (1126–2674)	1476 (963–2413)	2403 (1974–3278)	<0.001 ^#^
SOFA	8 (6–10)	7 (6–9)	9 (8–11)	0.008 ^#^
ESR mm	39 (27–52)	38 (28–51)	41 (27–54)	ns ^#^
CRP (mg/L)	129 (106–137)	123 (95–135)	132 (120–140)	<0.001 ^#^
GCS	12 (10–14)	13 (12–14)	11 (10–13)	<0.001 ^#^
Creatinine	1.33 (0.97–2.31)	1.21 (0.99–2.24)	1.82 (0.96–2.46)	ns ^#^
Bilirubin	1.2 (1.0–2.0)	1.2 (1.0–2.0)	1.3 (1.1–2.1)	ns ^#^
Lactate	1.4 (1.0–2.3)	1.1 (1.0–1.4)	3.0 (2.3–3.9)	<0.001 ^#^
Death (%)	34.2%	23.7%	55.3%	0.035 *

## Data Availability

Not applicable.
